# *Lnc-C/EBPβ* Modulates Differentiation of MDSCs Through Downregulating IL4i1 With C/EBPβ LIP and WDR5

**DOI:** 10.3389/fimmu.2019.01661

**Published:** 2019-07-17

**Authors:** Yunhuan Gao, Wencong Shang, Dan Zhang, Shiwu Zhang, Xipeng Zhang, Yuan Zhang, Rongcun Yang

**Affiliations:** ^1^State Key Laboratory of Medicinal Chemical Biology, Nankai University, Tianjin, China; ^2^Key Laboratory of Bioactive Materials Ministry of Education, Nankai University, Tianjin, China; ^3^Department of Immunology, Nankai University School of Medicine, Nankai University, Tianjin, China; ^4^Department of Pathology, Tianjin Union Medical Center, Tianjin, China; ^5^Department of Colorectal Surgery, Tianjin Union Medical Center, Tianjin, China

**Keywords:** MDSC, lncRNA, IL4il, WDR5, H3K4me3

## Abstract

Myeloid-derived suppressor cells (MDSCs), which play an important role in tumor and inflammatory diseases, are divided into two subsets CD11b^+^Ly6C^hi^Ly6G^−^ monocytic MDSC (Mo-MDSC) and CD11b^+^Ly6C^low/neg^Ly6G^+^ polymorphonuclear MDSC (PMN-MDSC) with different immunosuppressive function. However, it is poorly understood the mechanism(s) to control differentiation of Mo-MDSCs and PMN-MDSCs. Here, we found that *lnc-C/EBP*β may promote PMN-MDSC but impede differentiation of Mo-MDSCs *in vitro* and *in vivo*. We demonstrated that *lnc-C/EBP*β mediated differentiation of MDSCs was through downregulating multiple transcripts such as IL4il. *Lnc-C/EBP*β not only bound to C/EBPβ isoform LIP to inhibit the activation of C/EBPβ but also interacted with WDR5 to interrupt the enrichment of H3K4me3 mark on the promoter region of IL4i1. Data also imply that conserved homo *lnc-C/EBP*β has a similar function with mouse *lnc-C/EBP*β. Since MDSC subsets exert different suppressive function, *lnc-C/EBP*β may be acted as a potential therapeutic target for inflammatory and tumor-associated diseases.

## Introduction

Myeloid-derived suppressor cells (MDSCs) have emerged as a major regulator of immune responses in cancer and other pathological conditions (1–4). In mice, MDSCs are broadly identified as CD11b^+^Gr1^+^cells. The Gr1^+^ subsets may be more accurately identified based on Ly6C and Ly6G markers as CD11b^+^Ly6C^hi^Ly6G^−^ monocytic MDSC (Mo-MDSC) and CD11b^+^Ly6C^low/neg(negative)^Ly6G^+^ polymorphonuclear MDSC (PMN-MDSC) (5, 6). Human MDSCs including monocytic (CD14^+^) MDSCs and PMN (CD15^+^) MDSCs, are described as lineage negative cells that co-express CD11b and CD33 but lack HLA-DR (7). These MDSCs play a pivotal role in cancer progression and other relative diseases by suppressing both innate and adaptive immune responses. However, recent studies have shown that Mo-MDSCs are more prominent than PMN-MDSCs. Mo-MDSCs may rapidly differentiate to tumor-associated macrophages (TAM) in tumor environment (2). Furthermore, Mo-MDSCs can produce large amounts of NO, Arg-1, and immune suppressive cytokines (2). Thus, it is necessary to understand the mechanism(s) to control the differentiation of Mo-MDSCs and PMN-MDSCs.

MDSCs are generated from common myeloid progenitor cells in bone marrow (BM). However, their development is governed by a complex network of signals including promoting accumulation of immature myeloid cells and providing for the pathological activation of these cells (2, 8). Inflammation and tumor-dependent activation of transcription factors, transcriptional co-regulators, and chromatin modifying factors collaborates to determine the differentiation of MDSCs (4, 7). Cytokines such as granulocyte-macrophage colony stimulating factor (GM-CSF) and interleukin (IL-6) could *in vitro* induce development of MDSCs. Transcription factors such as CCAAT/enhancer binding protein β (C/EBPβ), phosphorylated signal transducer of activator of transcription 3 (phospho-STAT3), and C/EBP homologous protein (CHOP) centrally regulate MDSC expansion (1, 4, 7, 9, 10). Recent studies show that epigenetic modification such as lncRNAs (length >200 nucleotides) also play an important role in regulating differentiation and function of immune cells. These lncRNAs can be intergenic (between protein coding genes), intronic, and natural antisense transcripts, or transcribed from divergent enhancers and promoters (11). They may regulate gene expression in diverse biological processes through binding to chromatin-modifying factors, transcription factors (12). Multiple lncRNAs have been described in myeloid derive cells such as *lnc-DC* in dendritic cells (13), *lincRNA-Cox2, lincRNA-EPS*, and *AS-IL-1a* in macrophages (14–16), *lncRNA-Morrbid* in myeloid cells (17). However, effect(s) of lncRNA on differentiation and function of MDSCs is very little understood.

Our previous studies have shown that inflammatory or tumor associated factors may affect the development of MDSCs (18), and also found that differentiation and function of MDSCs may be regulated through microRNA(s), lncRNA and epigenetic modifying factors (19, 20). We here found that *lnc-C/EBP*β, which was identified by us (21), had a novel role in the differentiation of MDSCs. We found that *lnc-C/EBP*β may promote PMN-MDSCs but impede the differentiation of Mo-MDSCs. We demonstrate that *lnc-C/EBP*β may downregulate IL4il to affect the differentiation of MDSCs through binding with *C/EBP*β *LIP* and *WDR5*. These may provide a novel target for controlling MDSC differentiation.

## Materials and Methods

### Mice, Human Samples, and Cell Lines

C57BL/6 mice were purchased from the Beijing Animal Center (Beijing, China) and maintained in a specific pathogen-free (SPF) facility. B6.SJL-CD45a (Ly5a) (CD45.1) mice were purchased from the Model Animal Research Center of Nanjing University (Nanjing, Jiangsu, China). All animal experiments were carried out in accordance with Nankai University Guide for the Care and Use of Laboratory Animals. The peripheral blood and tissues samples from the patients and healthy human were obtained after signing informed consent at People Union Hospital (Tianjin, China). The collection and use of all human samples were approved by the Institute's Human Ethics Committee of Nankai University and in accordance with the Declaration of Helsinki.

Murine melanoma B16, breast cancer 4T1, colon cancer CT26, and human embryonic kidney cell line HEK 293T cells were obtained from the American Type Culture Collection (ATCC, USA). Murine ovarian tumor cell line 1D8 was from Dr. Richard Roden (The Johns Hopkins University School of medicine, USA).

### Reagents

Recombinant murine GM-CSF and IL-6 were purchased from PeproTech (Rocky Hill, NJ). Mouse anti-C/EBPβ, anti-IL4i1, and anti-H3K4me3 were from Abcam (Cambridge, MA). Rabbit anti-WDR5 was from Cell Signaling Technology (Beverly, MA). Mouse anti-V5 was purchased from Thermo Fisher Scientific (Pittsburgh, PA). Anti-Gr1-PE, anti-CD11b-PerCP/Cy5.5, anti-Ly6G-PE, anti-Ly6C-FITC, anti-F4/80-FITC, anti-CD3-PerCP/Cy5.5, anti–CD45-APC, anti–CD45.1-Cy7, anti-CD33-PE, anti-HLA-DR-APC, anti-CD14-FITC, anti-CD15-PE were from BD Biosciences (San Diego, CA).

### siRNAs, Lentiviruses, and Plasmid Construction

siRNAs were from Riobio (Guangzhou, China). siRNA sequences for *IL4i1*and *WDR5* were listed in Supplementary Table S1. ShRNA targets were chosen from the target sequences produced by BLOCK-iT™ RNAi Designer (Invitrogen) and/or by i-Score Designer. ShRNA constructs were made using pGreenPuro™ shRNA cloning and expression lentivector kit (System Biosciences Inc., USA) according to the manual. Control shNC was luciferase control shRNA from the kit. For packaging of lentivirus particles, shRNA lentivectors or *lnc-C/EBP*β lentivectors together with pMD2G and psPAX2 packaging plasmids were co-transfected into 293T cells. The full-length sequences of C/EBPβ LAP/LIP, IL4i1, and WDR5 were amplified using PCR (Primer pairs used were described in Supplementary Table S1). The PCR products were cloned into the pcDNA™3.1/V5-His TOPO® TA plasmid (Invitrogen, USA). The lentiviruses, siRNAs or plasmids were used to transduce or transfect MDSCs.

### Transduction and Transfection

BMCs were collected from C57BL/6 mice and cultured in six-well plate. Human monocytes were collected and isolated from peripheral blood. For transduction, the cells were infected with shRNA lentivectors [50 multiplicity of infection (MOI)] or *lnc-C/EBP*β lentivectors (50 MOI) or control empty lentiviruses (50 MOI) in the presence of 8 μg/ml polybrene (Millipore, USA) by centrifugation and then cultured with complete medium for 24 h. For transfection, the cells were transfected with WDR5 siRNA (100 nM), IL4il siRNA (100 nM), negative control siRNA (100 nM) or pcDNA3.1/WDR5 (4 μg/ml), pcDNA3.1/IL4il (4 μg/ml), and empty pcDNA3.1 control (4 μg/ml) using Lipofectamine™ 3000 (Invitrogen, USA) or HiPerFect transfection reagent (siRNA transfection) (Qiagen, USA) according to the manufacturer's instructions. The cells were then washed and cultured under GM-CSF or GM-CSF plus IL-6 or tumor supernatants for 4 days.

### Generation of Inflammatory and Tumor Associated MDSCs

Inflammatory factors associated MDSCs were generated by culturing bone marrow cells of C57BL/6 mice in flask or 6-well plate for 4 days in the presence of 5% FBS medium containing GM-CSF (40 ng/ml) only or GM-CSF (40 ng/ml) plus IL-6 (40 ng/ml) (20). To prepare tumor cell supernatant-induced CD11b^+^Gr1^+^ MDSCs *in vitro*, 5 × 10^4^ 1D8, 4T1, CT26, or B16 tumor cells (upper chamber) were co-cultured with 2 × 10^6^ BMCs (lower chamber) in a 24-transwell plate in the presence of GM-CSF (40 ng/ml) for 4 days. Human MDSC-like cells were generated according to previous reported methods (22).

### Microarray

Microarray of coding mRNA in *lnc-C/EBP*β knockdown MDSC was performed using Affymetrix GeneChip mouse Genome 430 2.0 array by Beijing Capitalbio Technology Co., Ltd. Briefly, total RNA was extracted using Trizol (Life Technologies). Contaminating DNAs were removed using RNeasy spin columns (Qiagen). The quality of isolated RNA samples was evaluated with an Agilent Bioanalyzer 2100 (Agilent technologies) and the purified RNA was quantified using a NanoDrop ND-2000 spectrophotometer (Infinigen Biotechnology Inc.). Microarray of coding mRNA in *lnc-C/EBP*β knockdown MDSC was performed using Affymetrix GeneChip mouse Genome 430 2.0 array. The R software (v.2.13.0) platform was applied to analyze the microarray data, and the limma (linear regression model) package was used to statistically analyze differentially expressed genes. The expression levels of mRNAs at each time point were compared with control. Genes having a fold change > 2 or < −2 and an adjusted *p* < 0.05 were considered as differentially expressed. Microarray GEO accession number GSE104571.

### Flow Cytometry

Cells were collected and rinsed twice with ice cold PBS, incubated with FITC-, PE-, percy5.5-, or APC-labeled antibodies for 30 min in PBS with 1% FBS according to our previous method (23). After washed twice, cells were resuspended in PBS and analyzed using a FACScan flow cytometer (BD Biosciences). Dead cells were eliminated through 7-AAD staining.

### RNA Extraction and qRT-PCR

Total RNA was extracted from the cells, tissues, and organs using TRIzol reagent (Invitrogen). First-strand cDNA was generated from total RNA using oligo-dT/random primer mix and reverse transcriptase (Invitrogen Corp). Quantitative real-time PCR (qRT-PCR) was conducted using QuantiTect SYBR Green PCR Master Mix (Qiagen) and specific primers in an ABI Prism 7000 analyzer (Applied Biosystems). GAPDH mRNA expression was detected in each experimental sample as an endogenous control. The fold changes were calculated using the ΔΔCt method according to the manufacturer's instructions (Applied Biosystems). All the reactions were run in triplicate.

### Western Blot Analyses

Cells were harvested at the indicated times and rinsed twice with ice cold PBS. Cell extracts were prepared with lysis buffer and centrifuged at 13,000 g for 10 min at 4°C. Protein samples were electrophoresed using 12% polyacrylamide gels and transferred to PVDF membranes. After the membranes were blocked with 5% skim milk powder for 1 h at room temperature, they were incubated with first antibody in TBST overnight at 4°C. Secondary antibodies with horseradish peroxidase (HRP) (1:5000) were labeled according to our previous method (23). The signals were checked by autoradiography film when HRP substrate was added to the membranes.

### Immunostaining and RNA Fluorescence *in situ* Hybridization

Immunostaining and RNA Fluorescence *in situ* hybridization (RNA-FISH) were performed according to reported protocol (24). Cells were first slicked on sterile and 0.01% poly-lysine-treated slides in the bottom of 6-well tissue culture dish. After that, the slides were processed sequentially with ice-cold CSK buffer, CSK+0.4% Triton X-100 buffer, and CSK buffer for 30 s for cell membrane perforation. The slides were then treated with 4% PFA for 10 min and cold 70% ethanol three times for cells fixation. After rinsed three times with ice cold PBS, the slides were blocked in pre-warmed 5% goat serum for 30 min at 37°C. Then, the slides were incubated with primary antibody at 37 °C for 1 h, washed three times with 1×PBS/0.2% Tween-20 for 3 min on a rocker, and then incubated with secondary antibody at 37°C for 30 min. After washing three times with 1×PBS/0.2% Tween-20, the slides were fixed with 2% PFA at room temperature for 10 min. The slides were dehydrated by moving them through a room temperature ethanol series (85, 95, and 100% ethanol) for 2 min each, and air-dried at room temperature for 15 min and hybridized using the indicated probes overnight at 37°C in a humid chamber. After washing with 2× SSC/50% formamide, 2× SSC, and 1× SSC each for three times, DAPI dye was added. Finally, the slides were sealed, and then observed using confocal microscope.

### Chromatin Immunoprecipitation-PCR

Chromatin immunoprecipitation (CHIP)-PCR was performed using EZ-CHIP™ chromatin immunoprecipitation kit (Millipore, USA) according to the reported method (25). MDSCs were crosslinked with 1% paraformaldehyde and incubated with rotation at room temperature. Crosslinking was stopped after 10 min with glycine to a final concentration of 0.125 M and incubated 5 min further with rotation. Cells were washed with ice cold PBS (containing 1% PMSF) 3 times and immediately resuspended in SDS lysis buffer (containing 1% PMSF). Cell lysates were sonicated for 40 cycles of 30 s ON and 30 s OFF in 10 cycle increments using a Biorupter (Diadenode) on ice. After pelleting debris, protein G agarose was added and incubated for 1 h at 4°C with rotation for preclearing. For immunoprecipitation, pre-cleared cell lysate was incubated with the indicated antibodies (1 μg per 2 million cells) overnight with the rotation at 4°C and protein G agarose was added for the final 2 h of incubation. Beads were washed with low salt, high salt, LiCl wash buffer, and chromatin immunocomplex was eluted using elution buffer through incubating at room temperature for 15 min. Reverse crosslinks of protein/DNA complexes to free DNA were realized through adding 5 M NaCl and incubating at 65°C overnight. qPCR was performed on DNA purified after treatment with RNase (30 min, 37°C) and proteinase K (2 h, 55°C) after reversal of crosslinks.

### RNA Immunoprecipitation (RIP)

RNA immunoprecipitation (RIP) was performed according to previously reported protocol (24). Briefly, the cells were harvested, washed, added ice cold IP lysis buffer (Thermo Scientific Pierce) containing 0.5% ribonuclease inhibitor (Invitrogen), and incubated on ice for 5 min with periodic mixing. Then the lysates were transferred into a microcentrifuge tube and centrifuged at 13,000 g for 10 min to pellet the cell debris at 4°C, and the supernatants were transferred into a new tube, and protein G agarose was added and incubated for 1 h at 4°C with rotation for preclearing. The immunoprecipitating antibody was added and incubated overnight at 4°C with rotation. Protein G agarose was pelleted by brief centrifugation (3,000 g for 1 min) and then washed sequentially with IP lysis buffer (containing 0.5% ribonuclease inhibitor). Finally, RNA was extracted from protein/RNA complexes on the beads using TRIzol reagent and dissolve in DEPC-water and quantified by qPCR.

### Immunoprecipitation-MASS

Immunoprecipitation (IP)-MASS was performed according to our previous method (20). Briefly, the MDSCs were lysed in IP lysis buffer (Pierce) containing 10% PMSF. Protein A/G magnetic beads (Pierce) were first added into the cell lysates for pre-clearing. The supernatants were collected after centrifuging at 12,000 rpm and then immunoprecipitated overnight at 4°C with the indicated antibodies. Protein A/G Magnetic Beads were added into cell lysates and incubated for additional 3 hrs. After being washed with IP lysis buffer for five times, Protein A/G Magnetic Beads were denatured and resolved by SDS-PAGE gels, and followed by silver staining. The gel lanes containing the immunopurified samples were excised for liquid chromate graphy-tandem MS (LC-MS/MS) analysis by Tsinghua University.

### RNA-Protein Pull-Down Analyses

RNA-protein pull-down analyses were performed using Pierce™ magnetic RNA-protein pull-down kit. MDSCs were harvested and cell lysates were prepared using IP lysis buffers (Thermo Scientific Pierce, USA) (26). *Lnc-C/EBP*β was transcribed (NEB, manual HiScribe T7 *in vitro* transcription Kit) and labeled using RNA 3′ Desthiobiotinylation Kit (Thermo Scientific Pierce) *in vitro*. 50 ul beads and 50 pmol of labeled RNA were added into RNA capture buffer, and incubated for 30 min at room temperature with agitation to binding of labeled *lnc-C/EBP*β to streptavidin magnetic beads. After washing beads with an equal volume of 20 mM Tris (pH 7.5), 100 μL of 1× protein-RNA binding buffer was added into the beads and mixed well. 100 μL of master mix of RNA-protein binding reaction was added to the RNA-bound beads, mixed by pipetting and then incubated 60 min at 4°C with rotation to binding of RNA-binding proteins to RNA. After washing beads with 100 ul wash buffer for twice, 50 μL of elution buffer was added, and incubated 30 min at 37°C with agitation. The samples were analyzed on a gel.

### Statistical Analyses

Statistical analyses were performed using two-tailed student's *t*-test and GraphPad Prism 5 software. A 95% confidence interval was considered significant and defined as *p* < 0.05. ^*^ indicates *p* < 0.05, ^**^p < 0.01, ^***^p < 0.001.

## Results

### Different Levels of *Lnc-C/EBPβ* Expression in PMN-MDSC, Mo-MDSC, and Macrophages

**Figure 1:**
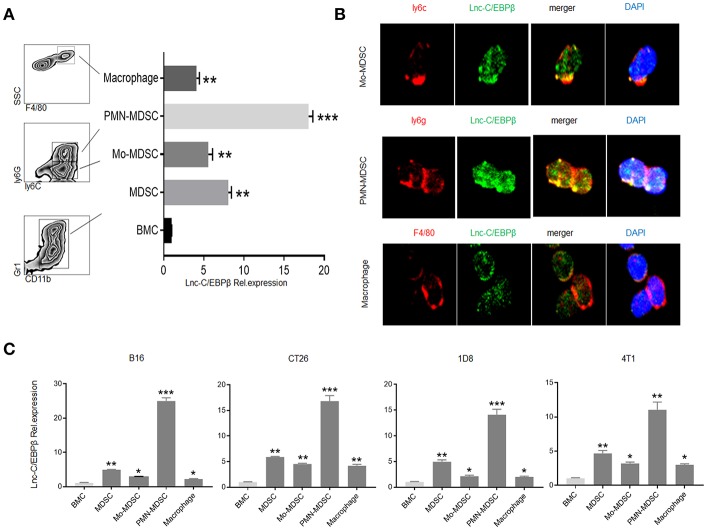
Different levels of *lnc-C/EBP*β in PMN-MDSCs, Mo-MDSCs, and macrophages. **(A)** QRT-PCR of *lnc-C/EBP*β in the MDSC subsets isolated from GM-CSF plus IL6 mediated MDSCs. **(B)** Fluorescence *in situ* hybridization of *lnc-C/EBP*β in mouse MDSC subsets. **(C)** QRT-PCR of mouse *lnc-C/EBP*β in MDSC subsets isolated from the tumor of mice bearing melanoma B16, colon cancer CT26, ovarian cancer 1D8, and breast cancer 4T1. Data in **(A,C)** are a representative of three independent measurements. Two-tailed, paired *T*-test was used in **(A,C)**. ^*^p < 0.05; ^**^p < 0.05; ^***^p < 0.001.

Our previous studies have shown that *lnc-C/EBP*β negatively regulates suppressive function of MDSCs (21). Since lncRNAs often exert multiple effects on the function and differentiation of the cells, we here investigated the role of *lnc-C/EBP*β in the differentiation of MDSCs. We first examined the expression levels of *lnc-C/EBP*β in different MDSC subsets. BMCs were cultured *in vitro* for 4 days in the presence of GM-CSF and IL-6. MDSC subsets were sorted by flow cytometry and analyzed using qRT-PCR, immunostaining, and RNA-FISH. Data showed that *lnc-C/EBP*β could be detected in PMN-MDSC, Mo-MDSC, and F4/80^+^macrophage. However, the level of *lnc-C/EBP*β in PMN-MDSCs was significantly higher than in Mo-MDSCs and F4/80^+^macrophages (Figures 1A,B). Importantly, isolated PMN-MDSCs from WT mice bearing melanoma B16, colon cancer CT26, ovarian cancer ID8, and breast cancer 4T1 had also higher levels of *lnc-C/EBP*β than in Mo-MDSCs and F4/80^+^macrophages (Figure 1C). These data indicate that there exist different levels of *lnc-C/EBP*β in PMN-MDSCs, Mo-MDSCs, and macrophages.

### *Lnc-C/EBPβ* Promotes PMN-MDSC but Impedes Differentiation of Mo-MDSC

**Figure 2:**
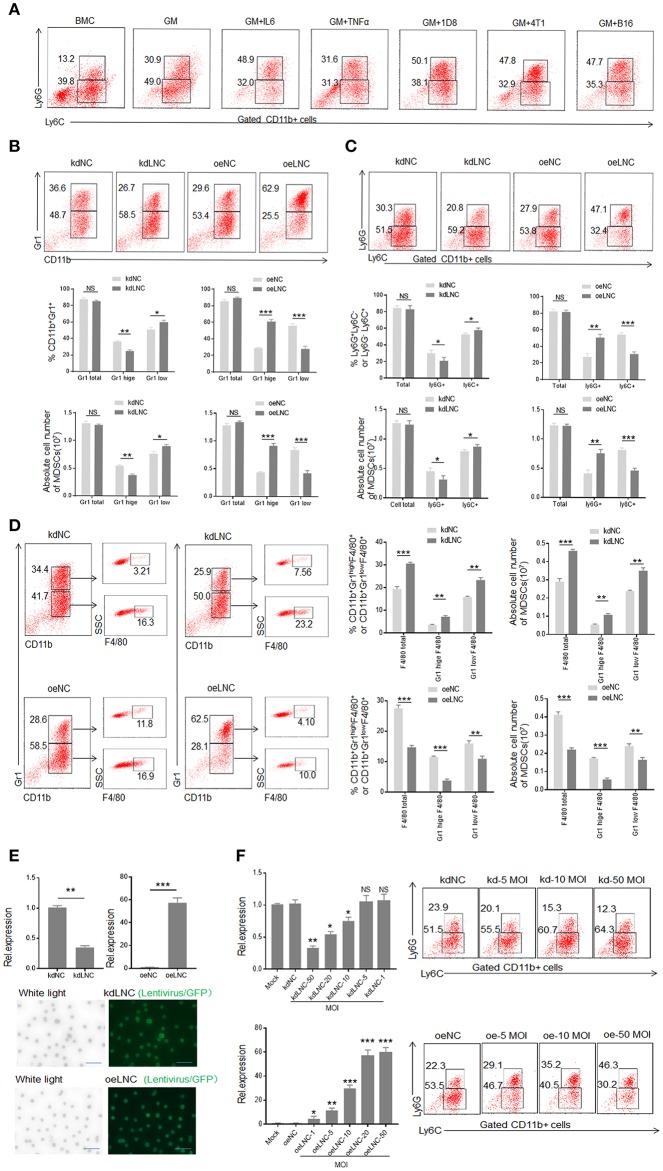
*Lnc-C/EBP*β promotes differentiation of PMN-MDSC but impedes Mo-MDSC *in vitro*. **(A)** Flow cytometry of inflammatory cytokine and tumor mediated CD11b^+^Ly6G^+^Ly6C^−^ and CD11b^+^Ly6G^−^Ly6C^+^ MDSCs. 1D8, mouse ovarian carcinoma; 4T1, mouse breast cancer; B16, mouse melanoma. BMC, bone marrow cells; GM, GM-CSF. **(B)** Flow cytometry of Gr1^+^CD11b^+^, Gr1^hi^CD11b^+^, and Gr1^low^CD11b^+^ MDSCs in *lnc-C/EBP*β knockdown or exogenous *lnc-C/EBP*β treated MDSCs. The percentages and absolute number of cells were compared (*n* = 3). **(C)** Flow cytometry of CD11b^+^Ly6G^+^Ly6C^−^ and CD11b^+^Ly6G^−^Ly6C^+^ MDSC in *lnc-C/EBP*β knockdown or exogenous *lnc-C/EBP*β treated MDSCs. The percentages and absolute number of cells were compared (*n* = 3). **(D)** Flow cytometry of *lnc-C/EBP*β nockdown and extinct *lnc-C/EBP*β treated MDSCs. After culturing for 4 days, *lnc-C/EBP*β knockdown and extinct *lnc-C/EBP*β treated MDSCs were analyzed by staining using anti-Gr-1, anti-CD11b, and F4/80 antibody. The percentages and absolute number of cells were compared (*n* = 3). **(E)** QRT-PCR of mouse *lnc-C/EBP*β in MDSCs after transfecting with *lnc-C/EBP*β lentivirus (upper) and fluorescence microscopic imaging of green fluorescent protein in lentivirus infected cells (lower). **(F)** QRT-PCR of mouse *lnc-C/EBP*β in cells transfected with different concentrations of lentivirus and flow cytometry of CD11b^+^ly6G^+^Ly6C^−^, CD11b^+^ly6G^−^Ly6C^+^ MDSCs in *lnc-C/EBP*β lentivirus treated MDSCs. MOI, multiplicity of infection; kdLNC(kd), lentivirus/*lnc-C/EBP*β shRNA; oeLNC(oe), lentivirus/*lnc-C/EBP*β; kdNC and oeNC, control lentiviruses. Data are a representative of at least three experiments. Absolute number = total cell number × percentage of MDSC subsets. Two-tailed, paired *T*-test was used in B-F. *p < 0.05; **p < 0.05; ^***^p < 0.001. NS, no significant.

**Figure 3:**
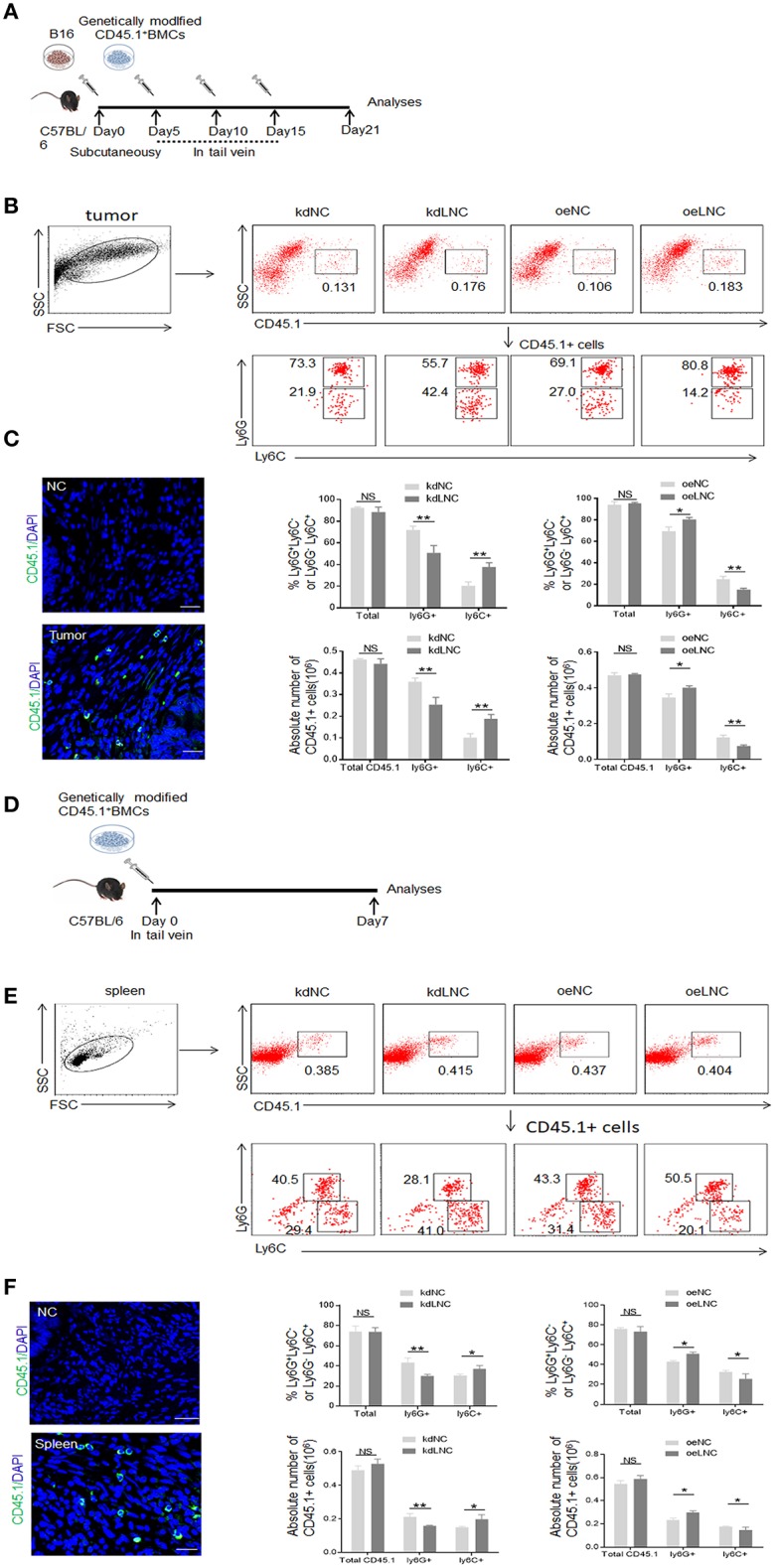
*Lnc-C/EBP*β promotes differentiation of PMN-MDSC but impedes Mo-MDSC *in vivo*. **(A)** Schematic of the experiment. *Lnc-C/EBP*β lentivirus treated CD45.1^+^ BMCs were injected into B16 tumor bearing WT mice in tail vein, and after 21 days the tumor CD45.1 cells were analyzed. **(B)** Flow cytometry of MDSCs in mouse tumor after injecting *lnc-C/EBP*β lentivirus treated CD45.1^+^BMCs. The percentage changes and absolute cell number of CD45.1^+^ cell of MDSC subsets were compared (*n* = 6). **(C)** Confocal microscopy of CD45.1^+^ cells in tumor. Green, CD45.1; blue, DAPI; NC, isotypic Ab. Scale bars = 100 μm. **(D)** Schematic of the experiment. *lnc-C/EBP*β lentivirus treated CD45.1^+^ BMCs were injected into WT mice in tail vein, and after 1 week the spleen CD45.1 cell was analyzed. **(E)** Flow cytometry of MDSCs in mouse spleen after injecting *lnc-C/EBP*β lentivirus treated CD45.1^+^MDSCs. The percentage changes and the absolute cell number of CD45.1^+^ MDSC subsets were compared (*n* = 6). **(F)** Confocal microscopy of CD45.1^+^ cells in spleen. Green, CD45.1; blue, DAPI; NC, isotypic Ab. Scale bars = 100 μm. kdLNC, lentivirus/*lnc-C/EBP*β shRNA; oeLNC, lentivirus/*lnc-C/EBP*β; kdNC and oeNC, control lentiviruses. Flow cytometry in **(B,E)** is a representative of at least three experiments. Absolute number = total CD45.1^+^ cell number in spleen or tumor × percentage of CD45.1^+^ MDSC subsets. Two-tailed, paired *T*-test was used in **(B,D)**. ^*^p < 0.05; ^**^p < 0.05; NS, no significant.

MDSCs could be induced by inflammatory cytokines and tumor cells (Figure 2A). Compared with GM-CSF alone, GM-CSF plus IL6 mediated MDSCs contained lower percentage of Ly6C^+^ cells (Figure 2A). Our previous studies found that the expression of *lnc-C/EBP*β may be regulated by IL 6, and *lnc-C/EBP*β may potentially promote the differentiation of PMN-MDSC (Ly6G^high^ CD11b^+^Ly6C^Low/neg^) (21). Consistent with this finding, *lnc-C/EBP*β knockdown inhibited BMCs to differentiate into Gr1^high^ CD11b^+^ MDSCs; whereas exogenous *lnc-C/EBP*β promoted the differentiation of Gr1^high^CD11b^+^MDSCs (Figure 2B), indicating that *lnc-C/EBP*β may promote the differentiation of PMN-MDSCs. *Lnc-C/EBP*β transduction decreased the fraction of CD11b^+^Ly6C^high^Ly6G^−^ Mo-MDSC subpopulation; whereas the percentages and absolute cell number of Mo-MDSCs increased in *lnc-C/EBP*β silencing bone marrow cells (BMCs) (Figure 2C), suggesting that *lnc-C/EBP*β not only promotes the differentiation of PMN-MDSCs but also impedes differentiation of MDSCs into Mo-MDSCs. Notably, F4/80^+^ macrophages significantly increased after *lnc-C/EBP*β knockdown. The exogenous *lnc-C/EBP*β also inhibited the differentiation of MDSCs into F4/80^+^ macrophages (Figure 2D). We further proved the validity of *lnc-C/EBP*β lentivirus by qRT-PCR and fluorescence microscopic imaging of green fluorescent protein in lentivirus infected cells (Figure 2E), and also excluded the effect of off-target by a dose-dependent experiment (Figure 2F), different shRNA to transfect BMCs and inflammatory responses (Supplementary Figures 1A–C). All of these indicate that *lnc-C/EBP*β may inhibit BMCs into Mo-MDSC/macrophages. To further confirm the effects of *lnc-C/EBP*β on the differentiation of MDSCs, we next employed a mouse CD45.1^+^ bone marrow cell (BMC) chimera model to investigate the effects of *lnc-C/EBP*β on MDSC differentiation *in vivo*. CD45.1^+^ BMCs from homogeneous mice were transduced with *lnc-C/EBP*β shRNA/lentiviruses and then injected into B16 tumor-bearing WT mice via tail vein at the indicated time (Figure 3A). Once mice were injected by exogenous *lnc-C/EBP*β transduced CD45.1^+^ BMCs, the proportion, and absolute cell number of CD11b^+^Ly6C^high^Ly6G^−^Mo-MDSC populations significantly decreased in the tumor-bearing mice; whereas increased Mo-MDSCs could be observed in those tumor bearing mice injected with *lnc-C/EBP*β knockdown CD45.1^+^ BMCs (Figure 3B). PMN-MDSCs increased in these mice injected with *lnc-C/EBP*β transduced CD45.1^+^ BMCs but decreased in the mice with *lnc-C/EBP*β knockdown CD45.1^+^ BMCs (Figure 3B). CD45.1^+^ cells could be detected in the tumor of mice injected by *lnc-C/EBP*β–transduced BMCs (Figure 3C), indicating successful establishment of the mouse model. To further confirm the effects of *lnc-C/EBP*β on the differentiation of BMCs, mice were also injected by exogenous *lnc-C/EBP*β transduced CD45.1^+^ BMC (Figure 3D). The proportion and absolute cell number of CD11b^+^Ly6C^high^Ly6G^−^Mo-MDSC populations significantly decreased in the spleen of mice; whereas increased Mo-MDSCs could be seen in the mice injected with *lnc-C/EBP*β knockdown CD45.1^+^ BMCs (Figure 3E). PMN-MDSCs increased in mice injected with *lnc-C/EBP*β transduced CD45.1^+^ BMC but decreased in mice with *lnc-C/EBP*β knockdown CD45.1^+^ BMC (Figure 3E). CD45.1^+^ cells could be detected in the spleen of mice injected by *lnc-C/EBP*β transduced BMCs (Figure 3F). Taken together, *lnc-C/EBP*β not only promotes the differentiation of PMN-MDSCs but also impedes MDSCs to Mo-MDSCs.

### *Lnc-C/EBPβ* Mediated Differentiation of MDSCs Is Through Downregulating Expression of Interleukin 4 Induced Gene-1

**Figure 4:**
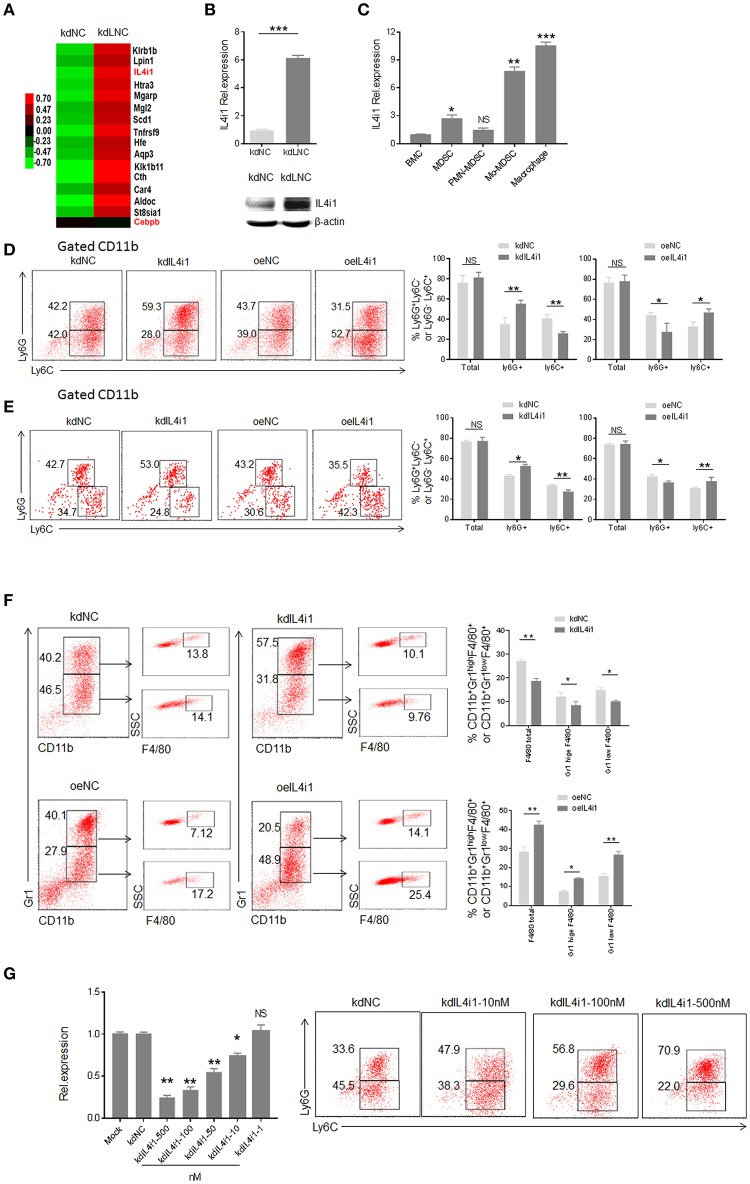
*lnc-C/EBP*β mediated differentiation of MDSCs depends on IL4il. **(A)** Gene expression in *lnc-C/EBP*β knockdown treated MDSC. **(B)** QRT-PCR (upper) and immunoblotting (lower) of IL4il in *lnc-C/EBP*β knockdown MDSCs. **(C)** QRT-PCR of IL4i1 in the MDSC subsets isolated from WT mice bearing B16 tumor. **(D)** Flow cytometry of CD11b^+^ly6G^+^Ly6C^−^, CD11b^+^ly6G^−^Ly6C^+^ MDSCs in IL4il knockdown cells or exogenous IL4i1 treated MDSCs. The percentages were compared (right, *n* = 3). **(E)** Flow cytometry of MDSCs in mouse spleen after injecting *IL4i1* knockdown or exogenous *IL4il* treated CD45.1^+^ BMCs. Percentage changes of CD45.1^+^ MDSC subsets was compared (right, *n* = 6). **(F)** Flow cytometry of IL4il siRNA or IL4il transfected MDSCs. After culturing for 4 days, IL4il siRNA or IL4il transfected MDSCs were analyzed by staining using anti-Gr-1, anti-CD11b, and F4/80 antibody. **(G)** QRT-PCR of mouse IL4i1 in cells transfected with different concentrations of siRNAs and flow cytometry of CD11b^+^ly6G^+^Ly6C^−^, CD11b^+^ly6G^−^Ly6C^+^ MDSCs in *IL4i1* knockdown treated *MDSCs*. kdIL4il, IL4il siRNA; oeIL4i1, IL4il plasmid; kdNC, siRNA control; oeNC, empty/pcDNA.3.1plasmid. Data in **(D–G)** are a representative of at least three experiments. Two-tailed, paired *T*-test was used in **(B–G)**. ^*^p < 0.05; ^**^p < 0.05; ^***^p < 0.001. NS, no significant. Also see “https://www.ncbi.nlm.nih.gov/geo/query/acc.cgi?acc=GSE104571”.

LncRNAs may exert its role through regulating gene expression (15, 27, 28). To find *lnc-C/EBP*β associated gene (s), which potentially affect the differentiation of MDSCs, we analyzed gene expression pattern of *lnc-C/EBP*β knockdown MDSCs using a microarray (Figure 4A). Data showed that *lnc-C/EBP*β knockdown affected multiple gene expression such as interleukin 4-induced gene-1(IL4i1). As shown (Figure 4A, https://www.ncbi.nlm.nih.gov/geo/query/acc.cgi?acc=GSE104571), IL4i1 was remarkably upregulated in *lnc-C/EBP*β knockdown MDSCs. The higher levels of expression could be further confirmed by qRT-PCR and immunoblot in these *lnc-C/EBP*β knockdown cells (Figure 4B). IL4i1 plays a critical role in the differentiation of monocyte/macrophages (29–31). Isolated PMN-MDSCs from WT mice bearing B16 tumor had much lower level of IL4i1 than Mo-MDSC and F4/80^+^ macrophages (Figure 4C), implying a potential role of IL4i1 in regulating the differentiation of MDSC subpopulations. We next used loss- and gain-of function studies to investigate the effects of IL4i1 on the differentiation of MDSCs. IL4i1 knockdown decreased the proportion of CD11b^+^ Ly6C ^high^Ly6G^−^ Mo-MDSC subpopulation; whereas the percentages of Mo-MDSC increased in exogenous IL4i1 transfected BMCs (Figure 4D). The proportion of CD11b^+^Ly6C^high^Ly6G^−^ Mo-MDSC populations significantly increased in the spleen of mice injected by exogenous IL4il transfected CD45.1^+^BMC. The increased CD11b^+^Ly6C^high^Ly6G^−^ Mo-MDSC could not be seen in the mice injected with IL4il knockdown CD45.1^+^ BMCs (Figure 4E). F4/80^+^ macrophages significantly decreased after *IL4i1* knockdown; whereas exogenous *IL4i1* promoted differentiation into F4/80^+^ macrophages (Figure 4F). The exogenous IL4i1 also inhibited the differentiation Ly6G^high^ CD11b^+^Ly6C^Low/neg^ PMN-MDSCs; but IL4il knockdown promoted the differentiation of PMN-MDSCs (Figures 4D–F). We further confirmed the validity of siRNA and excluded the effect of off-target by using different kinds and doses of siRNAs to transfect BMCs (Figure 4G, Supplementary Figure 1D). Thus, IL4il promotes the differentiation of Mo-MDSCs but impedes MDSCs into PMN-MDSCs. Since *lnc-C/EBP*β may downregulate the expression of IL-4il, these data indicate that IL4il is involved in *lnc-C/EBP*β mediated differentiation of MDSCs.

### Binding of *Lnc-C/EBPβ* With C/EBPβ Isoform LIP and WDR5 Is Required for *Lnc-C/EBPβ* Mediated Downregulation of IL4il

**Figure 5:**
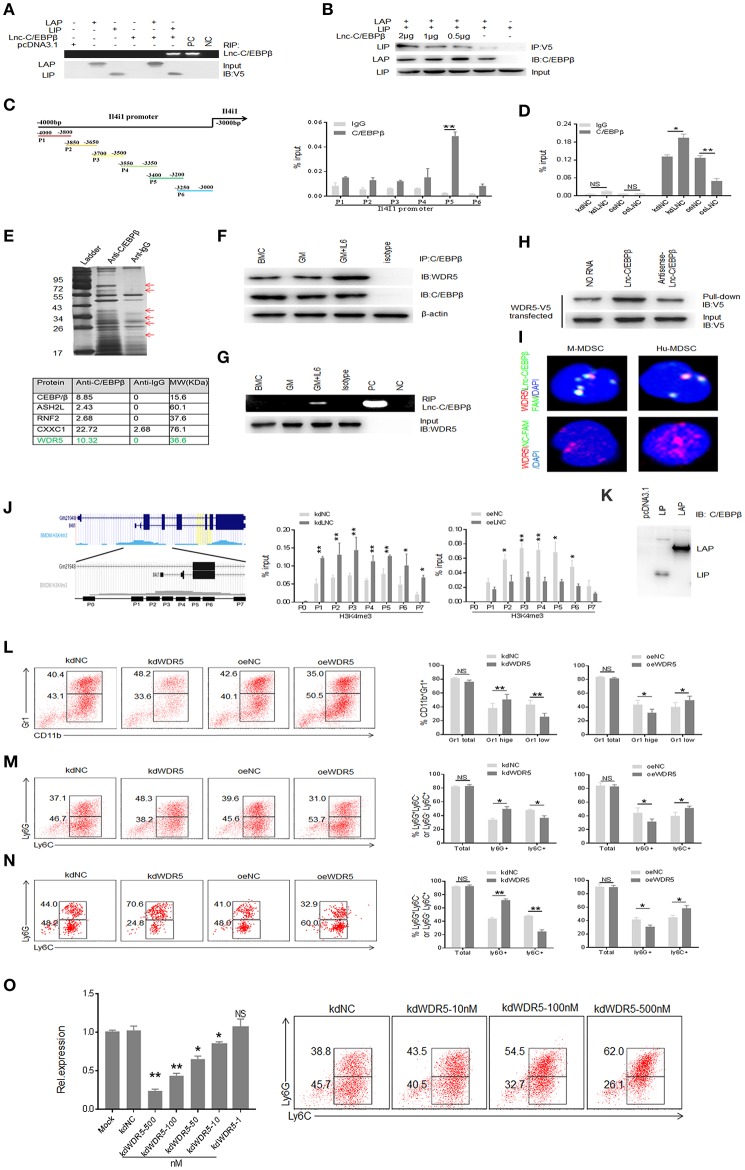
Binding of *lnc-C/EBP*β with C/EBPβ isoform LIP and WDR5 is required for *Lnc-C/EBP*β mediated Differentiation of MDSC. **(A)** RIP in V5-tagged LAP or LIP and lnc-C/EBPβ cotransfected HEK293T cells. RIP was performed using anti-V5 antibody and then PCR for lnc-C/EBPβ. pcDNA3.1, control plasmid; PC, positive control; NC, water. **(B)** Immunoblot of LAP and LIP in HEK293T cells. LIP/pcDNA3.1 (without V5-tagged), V5-tagged LAP/pcDNA3.1, and different concentrations of lnc-C/EBPβ pcDNA3.1 plasmids were cotransfected into 293T cells. IP was performed using V5 antibodies, and then immunoblot using anti-C/EBPβ (specific for both LAP and LIP). **(C,D)** CHIP-PCR on the promoter of IL4il of MDSCs. **(E)** IP-MASS of C/EBPβ interacting proteins (upper); Five C/EBPβ interacting partners associated with WDR5 were listed (lower). **(F)** Immunoblot of C/EBPβ and WDR5 interaction in GM-CSF alone and GM-CSFplusIL-6 mediated MDSCs. **(G)** RIP-PCR for *lnc-C/EBP*β used WDR5 antibody in differently generated MDSCs. **(H)** RNA-protein pull-down in *lnc-C/EBP*β and WDR5-V5 co-transfected HEK293T cells. No RNA and antisense RNA as control. **(I)** Immunostaining and RNA-FISH in mouse MDSC (M-MDSC, left) and human monocytes (Hu-MDSC, right). Red, WDR5; Green, *lnc-C/EBP*β; Blue, nuclei. **(J)** CHIP-PCR in the H3K4me3 enrichment region on the promoter of IL4il. **(K)** Immunoblotting of LAP and LIP in transfected HEK293T cells using anti-C/EBPβ antibody. 293T cells did not express LAP and LIP. **(L)** Flow cytometry of Gr1^+^CD11b^+^, Gr1^hi^CD11b^+^, and Gr1^low^CD11b^+^ MDSCs in *WDR5* knockdown or exogenous *WDR5* treated MDSCs. The percentages were compared (*n* = 3). **(M)** Flow cytometry of CD11b^+^Ly6G^+^Ly6C^−^ and CD11b^+^Ly6G^−^Ly6C^+^ MDSC in *WDR5* knockdown or exogenous *WDR5* treated MDSCs. The percentages were compared (*n* = 3). **(N)** Flow cytometry of MDSCs in mouse spleen after injecting *WDR5* knockdown or exogenous *WDR5* treated CD45.1^+^ BMCs. Percentage changes of MDSC subsets was compared (*n* = 6). **(O)** QRT-PCR of mouse WDR5 in cells transfected with different concentrations siRNA and flow cytometry of CD11b^+^ly6G^+^Ly6C^−^, CD11b^+^ly6G^−^Ly6C^+^ MDSCs in *WDR5* knockdown treated *MDSCs*. kdNC, siRNA control; kdWDR5, WDR5 siRNA; oeNC, control empty plasmid; oeWDR5, WDR5 plasmid. Error bars in **(C,D,J,L–O)** represent standard deviations from 3 independent measurements. Two-tailed, paired *T*-test was used in **(C,D,J,L–O)**. ^*^p < 0.05; ^**^p < 0.05; NS, no significant.

We next sought to determine underlying molecular mechanism(s) by which *lnc-C/EBP*β regulates expression of IL4il, which impedes the differentiation of Mo-MDSC. Consistent with previous findings (21), *lnc-C/EBP*β could bind with C/EBPβ isoform LIP to impede the activation of C/EBPβ (Figures 5A,B). Genome browser images showed that there had the binding site of C/EBPβ in the 5′ region of IL4il (Figure 5C). In the *lnc-C/EBP*β knockdown cells, CHIP-qPCR showed the increased C/EBPβ; whereas extrinsic *lnc-C/EBP*β reduced the enrichment of C/EBPβ in the 5′ region of IL4il (Figure 5D). All of these support that binding of *lnc-C/EBP*β with C/EBPβ isoform LIP may be involved in the regulation of IL4il gene.

Epigenetic modification plays a critical role in the gene expression such as that lysine methylation in the histones regulates the activation and repression of transcription (32). We next used *C/EBP*β as a bait to investigate whether *lnc-C/EBP*β also bind with other molecules, which are related to epigenetic modification. IP-MASS analyses showed that there were remarkable differences not only in the nuclear proteins but also in their signal molecule networks between GM-CSF and GM-CSF plus IL-6 mediated MDSCs (Figure 5E, Supplementary Figure 2). Data showed that WDR5 was a C/EBPβ targeting molecule (Figures 5E–K) and confirmed by co-immunoprecipitation (Figure 5F). Importantly, RIP and pull-down also confirmed the binding of *lnc-C/EBP*β with WDR5 (Figures 5G,H). The interaction of WDR5 with *lnc-C/EBP*β was also detected by FISH and immunostaining (Figure 5I). Thus, *lnc-C/EBP*β may bind with epigenetic factor WDR5. Since WDR5 is responsible for depositing the H3K4me3 mark on the promoter of active genes (33) and positively correlated with transcriptional activity, it also is possible for the binding of *lnc-C/EBP*β with epigenetic component WDR5 to promote gene expression. Genome browser images showed there also has the binding site of H3K4me3 mark in the 5′ region of IL4il. Using CHIP-qPCR, we investigated the effects of *lnc-C/EBP*β on the enrichment of H3K4me3 mark. In the *lnc-C/EBP*β knockdown cells, CHIP-qPCR showed the increased enrichment of H3K4me3 mark; whereas exogenous *lnc-C/EBP*β reduced this enrichment in the 5′ region of IL4il (Figure 5J). Therefore, the binding of *lnc-C/EBP*β with WDR5 affects the enrichment of H3K4me3 mark on the IL4il promoter and ultimately affect the expression of IL4i1. Further studies exhibited that WDR5 was indeed involved in the differentiation of MDSCs. WDR5 knockdown promoted the fraction of PMN-MDSC subpopulation; whereas the percentages of Mo-MDSCs increased in exogenous WDR5 transfected CD45.1^+^BMCs *in vitro* and *in vivo* (Figures 5L–N). We also further confirmed the validity of WDR5 siRNA, and excluded the effect of off-target by using different kinds and doses of siRNAs to transfect MDSCs (Figure 5O, Supplementary Figure 1E). Notably, other transcription factors and regulators such as *STAT3, STAT5, S100A8/A9, E2F3, IRF8* etc. which play an important role in the expansion and differentiation of MDSCs (1) were also potentially modulated by *lnc-C/EBP*β (Supplementary Figure 3A, https://www.ncbi.nlm.nih.gov/geo/query/acc.cgi?acc=GSE104558). H3K4me3 mark in the 5′ promoter region of these genes was remarkable enrichment (Supplementary Figure 3B), implying that the modulation of *lnc-C/EBP*β on these genes also is through WDR5. Taken together, *lnc-C/EBP*β may promote PMN-MDSCs but impede differentiation of Mo-MDSCs through interrupting the expression of differentiation associated genes such as IL4il.

### Human *Lnc-C/EBPβ* Has a Similar Function With Murine *Lnc-C/EBPβ*

**Figure 6:**
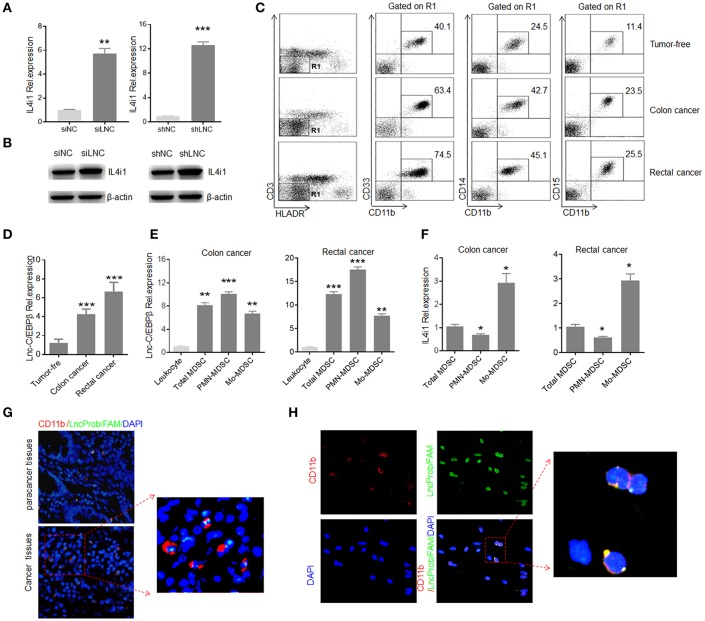
Human *lnc-C/EBP*β has a similar function with mouse. **(A)** QRT-PCR of *IL4i1* in *lnc-C/EBP*β knockdown human MDSC-like cells. **(B)** Immunoblotting of *IL4i1* in *lnc-C/EBP*β knockdown human MDSC-like cells. **(C)** Flow cytometry of Lin^−^HLA-DR^−^CD14^+^CD11b^+^ MDSCs subsets in healthy individuals and patients with colon and rectal cancer. **(D)** QRT-PCR of human *lnc-C/EBP*β in MDSC subsets isolated from the human colon, rectal cancer and healthy individuals. **(E)** QRT-PCR of human *lnc-C/EBP*β in MDSC subsets isolated from the human colon and rectal cancer. **(F)** QRT-PCR of human IL4i1 in MDSC subsets isolated from the human colon and rectal cancer. **(G)** Immunostaining and RNA-FISH in the human colon cancer and para cancer tissues. Red, CD11b; Green, *lnc-C/EBP*β*;* Blue, nuclei. One representative of 12 patients with colitic cancer. Scale bar = 200 μM. **(H)** Immunostaining and RNA-FISH in the peripheral blood Lin^−^HLA-DR^−^CD33^+^CD11b^+^ MDSCs in the patients with colon cancer. Red, CD11b; Green, *lnc-C/EBP*β; Blue, nuclei. One representative of 12 patients with colitic cancer. Scale bar = 200 μM. siNC, siRNA control; siLNC, *lnc-C/EBP*β siRNA; shNC, empty lentiviruses; shLNC, *lnc-C/EBP*β shRNA/lentiviruses. Error bars in **(A,D–F)** represent standard deviations from 3 independent measurements. Two-tailed, paired *T*-test was used in **(A,D–F)**. ^*^p < 0.05; ^**^p < 0.05; ^***^p < 0.001. NS, no significant.

Since human *lnc-C/EBP*β is high homology with mouse *lnc-C/EBP*β (21), we examined whether human *lnc-C/EBP*β had also similar function with mouse *lnc-C/EBP*β. Human MDSC-like cells were generated from human peripheral blood monocytes (PBMCs) after co-culturing with GM-CSF plus tumor supernatants or IL-6 (22). Interestingly, IL4il was also detected in these human MDSC-like cells. Furthermore, *lnc-C/EBP*β could modulate the expression of *IL4i1* (Figures 6A,B). To examine the modulation of *lnc-C/EBP*β on IL4il *in vivo* MDSCs, we isolated the MDSCs from patients with colorectal cancer, and examined the relationship between the expression of *lnc-C/EBP*β and IL4il. Lin^−^ HLA-DR^−^ CD33^+^ CD11b^+^ MDSCs were isolated from the blood of patients with glioblastoma, breast cancer, colon cancer, lung cancer and kidney cancer (34). Consistent with others (35–37), CD11b^+^CD14^+^ Mo-MDSC and Lin^−^HLA-DR^−^CD11b^+^CD15^+^ PMN-MDSC populations were much higher in patients with tumor as compared to tumor free-individuals (Figure 6C). Higher levels of *lnc-C/EBP*β could be detected in Lin^−^HLA-DR^−^ CD11b^+^ cells isolated from peripheral blood of patients with colorectal cancer as compared to Lin^−^HLA-DR^−^CD11b^+^ cells from tumor free healthy individuals (Figure 6D). QRT-PCR showed that there indeed existed negative relationship between *lnc-C/EBP*β and IL-4il in different MDSC subpopulations (Figures 6E,F). Finally, we also detected the expression of *lnc-C/EBP*β in the samples of patients with colorectal cancer. FISH and immuno-staining of colitis/carcinoma tissues demonstrated the expression of *lnc-C/EBP*β in CD11b^+^ monocytes/macrophages (Figure 6G) and nuclear localization of *lnc-C/EBP*β in the mononuclear cells of colitis and carcinoma (Figure 6H). Since *lnc-C/EBP*β may regulate the expression of MDSCs-associated genes *in vitro*, a high level of *lnc-C/EBP*β may play a role in the differentiation and function of MDSCs in patients with colorectal cancer. Thus, human *lnc-C/EBP*β is also involved in the differentiation of human MDSCs.

## Discussion

Previous data have already shown that transcription factor C/EBPβ plays a central role in regulating the differentiation and immune suppressive function of MDSCs (7, 38), and that WDR5 is responsible for depositing H3K4 methylation mark on the promoter region of active genes (33). We here demonstrate that the binding of *lnc-C/EBP*β with C/EBPβ and WDR5 affects the activity of C/EBPβ and enrichment of H3K4me3 to promote PMN-MDSCs but inhibit the differentiation of Mo-MDSCs. As *lnc-C/EBP*β is present in human monocyte-derived cells, it may be acted as a potential therapeutic target for inflammatory and tumor-associated diseases.

Our data show that *lnc-C/EBP*β may bind with C/EBPβ and WDR5 to affect differentiation of MDSCs through regulating expression of IL4il. IL4i1, a regulator of M2 macrophage polarization, may contribute to the regulation of monocyte/macrophage programming (29, 31, 39). C/EBPβ has been implicated in the differentiation of Mo-MDSCs (38, 40). Its deficiency affects mostly the differentiation of Mo-MDSCs (40). Mice lacking C/EBPβ in the hematopoietic system have lower frequencies of splenic Mo-MDSCs in MCA203 fibrosarcoma-bearing mice. Indeed, while the interaction of *lnc-C/EBP*β with C/EBPβ LIP affects LAP activity, MDSCs may not differentiate into Mo-MDSCs. WDR5 is involved in multiple biological processes and cell differentiation. It may mediate self-renewal and reprogramming of embryonic stem cell (41), and regulate immune cells through histone H3K4me3 (42, 43). Others also found that WDR5 binds with lncRNA HOTTIP to drive histone H3K4me3 and gene transcription (44).

That *lnc-C/EBP*β impedes MDSCs to differentiate into Mo-MDSC may have an important significance in the inflammation and tumor. Multiple reports have described the presence of MDSCs in patients with tumors such as colon cancer, lung cancer, breast cancer, pancreatic adenocarcinomas, urothelial carcinoma, kidney Cancer, glioblastoma (2, 34). Studies have shown that Mo-MDSCs have higher suppressive activity than PMN-MDSCs (38). In the tumor, Mo-MDSCs are more prominent than PMN-MDSC, and furthermore Mo-MDSCs may rapidly differentiate to tumor-associated macrophages (TAM) (2). These cells can produce large amounts of NO, Arg-1, and immune suppressive cytokines (2), which have much longer half-life than ROS produced by PMN-MDSCs (2, 38). Less immunosuppressive function of PMN-MDSCs than Mo-MDSCs is also confirmed in a single cell (34). In addition, PMN-MDSCs also have a short half-life (45). Thus, our data provide a new insight that will help direct development of novel immunotherapeutic methods for tumor through modulating the expression of *lnc-C/EBP*β to control differentiation of MDSCs into Mo-MDSCs.

## Data Availability

The datasets generated for this study can be found in Gene Expression Omnibus, GSE104571 GSE104558.

## Ethics Statement

All animal studies were conducted according to the Institutional Animal Care and Use Committee of the Model Animal Research Center. Animal experiments were approved by the Institute's Animal Ethics Committee of Nankai University.

## Author Contributions

RY designed the research and wrote the paper. YG conducted *in vivo* and *in vitro* experiments and immunoassay, participated in the study design, and performed the statistical analysis. WS was involved *in vitro* and *in vivo* assay. DZ, XZ, and SZ treated patient's samples. YZ offered assistances for the animal experiments. All authors read and approved the final manuscript.

### Conflict of Interest Statement

The authors declare that the research was conducted in the absence of any commercial or financial relationships that could be construed as a potential conflict of interest.

## References

[1] Bronte V Brandau S Chen SH Colombo MP Frey AB Greten TF. Recommendations for myeloid-derived suppressor cell nomenclature and characterization standards. Nat Commun. (2016) 7:12150. 10.1038/ncomms12150 27381735 PMC4935811

[2] Kumar V Patel S Tcyganov E Gabrilovich DI. The nature of myeloid-derived suppressor cells in the tumor microenvironment. Trends Immunol. (2016) 37:208–20. 10.1016/j.it.2016.01.004 26858199 PMC4775398

[3] Talmadge JE Gabrilovich DI. History of myeloid-derived suppressor cells. Nat Rev Cancer. (2013) 13:739–52. 10.1038/nrc3581 24060865 PMC4358792

[4] Ugel S De Sanctis F Mandruzzato S Bronte V. Tumor-induced myeloid deviation: when myeloid-derived suppressor cells meet tumor-associated macrophages. J Clin Invest. (2015) 125:3365–76. 10.1172/JCI80006 26325033 PMC4588310

[5] Youn JI Nagaraj S Collazo M Gabrilovich DI. Subsets of myeloid-derived suppressor cells in tumor-bearing mice. J Immunol. (2008) 181:5791–802. 10.4049/jimmunol.181.8.5791 18832739 PMC2575748

[6] Movahedi K Guilliams M Van den Bossche J Van den Bergh R Gysemans C Beschin A. Identification of discrete tumor-induced myeloid-derived suppressor cell subpopulations with distinct T cell-suppressive activity. Blood. (2008) 111:4233–44. 10.1182/blood-2007-07-099226 18272812

[7] Gabrilovich DI Nagaraj S. Myeloid-derived suppressor cells as regulators of the immune system. Nat Rev Immunol. (2009) 9:162–74. 10.1038/nri2506 19197294 PMC2828349

[8] Youn JI Collazo M Shalova IN Biswas SK Gabrilovich DI. Characterization of the nature of granulocytic myeloid-derived suppressor cells in tumor-bearing mice. J Leukocyte Biol. (2012) 91:167–81. 10.1189/jlb.0311177 21954284 PMC3250305

[9] Sonda N Simonato F Peranzoni E Cali B Bortoluzzi S Bisognin A. miR-142-3p prevents macrophage differentiation during cancer-induced myelopoiesis. Immunity. (2013) 38:1236–49. 10.1016/j.immuni.2013.06.004 23809164

[10] Thevenot PT Sierra RA Raber PL Al-Khami AA Trillo-Tinoco J Zarreii P. The stress-response sensor chop regulates the function and accumulation of myeloid-derived suppressor cells in tumors. Immunity. (2014) 41:389–401. 10.1016/j.immuni.2014.08.015 25238096 PMC4171711

[11] Ulitsky I Bartel DP. lincRNAs: genomics, evolution, and mechanisms. Cell. (2013) 154:26–46. 10.1016/j.cell.2013.06.020 23827673 PMC3924787

[12] Morris KV Mattick JS. The rise of regulatory RNA. Nat Rev Genet. (2014) 15:423–37. 10.1038/nrg3722 24776770 PMC4314111

[13] Wang P Xue Y Han Y Lin L Wu C Xu S. The STAT3-binding long noncoding RNA lnc-DC controls human dendritic cell differentiation. Science. (2014) 344:310–3. 10.1126/science.1251456 24744378

[14] Carpenter S Aiello D Atianand MK Ricci EP Gandhi P Hall LL. A long noncoding RNA mediates both activation and repression of immune response genes. Science. (2013) 341:789–92. 10.1126/science.1240925 23907535 PMC4376668

[15] Atianand MK Hu W Satpathy AT Shen Y Ricci EP Alvarez-Dominguez JR. A long noncoding RNA lincRNA-EPS acts as a transcriptional brake to restrain inflammation. Cell. (2016) 165:1672–85. 10.1016/j.cell.2016.05.075 27315481 PMC5289747

[16] Chan J Atianand M Jiang Z Carpenter S Aiello D Elling R. Cutting edge: a natural antisense transcript, AS-IL1alpha, controls inducible transcription of the proinflammatory cytokine IL-1alpha. J Immunol. (2015) 195:1359–63. 10.4049/jimmunol.1500264 26179904 PMC4530055

[17] Kotzin JJ Spencer SP McCright SJ Kumar DBU Collet MA Mowel WK. The long non-coding RNA Morrbid regulates Bim and short-lived myeloid cell lifespan. Nature. (2016) 537:239–43. 10.1038/nature19346 27525555 PMC5161578

[18] Yang R Cai Z Zhang Y Yutzy WH Roby KF Roden RB. CD80 in immune suppression by mouse ovarian carcinoma-associated Gr-1+CD11b+ myeloid cells. Cancer Res. (2006) 66:6807–15. 10.1158/0008-5472.CAN-05-3755 16818658

[19] Zhang M Liu Q Mi S Liang X Zhang Z Su X. Both miR-17-5p and miR-20a alleviate suppressive potential of myeloid-derived suppressor cells by modulating STAT3 expression. J Immunol. (2011) 186:4716–24. 10.4049/jimmunol.1002989 21383238

[20] Xin J Zhang Z Su X Wang L Zhang Y Yang R. Epigenetic component p66a modulates myeloid-derived suppressor cells by modifying STAT3. J Immunol. (2017) 198:2712–20. 10.4049/jimmunol.1601712 28193828

[21] Gao Y Sun W Shang W Li Y Zhang D Wang T. Lnc-C/EBPbeta negatively regulates the suppressive function of myeloid-derived suppressor cells. Cancer Immunol Res. (2018) 6:1352–63. 10.1158/2326-6066.CIR-18-0108 30171135

[22] Mace TA Ameen Z Collins A Wojcik S Mair M Young GS. Pancreatic cancer-associated stellate cells promote differentiation of myeloid-derived suppressor cells in a STAT3-dependent manner. Cancer Res. (2013) 73:3007–18. 10.1158/0008-5472.CAN-12-4601 23514705 PMC3785672

[23] Su X Min S Cao S Yan H Zhao Y Li H. LRRC19 expressed in the kidney induces TRAF2/6-mediated signals to prevent infection by uropathogenic bacteria. Nature Commun. (2014) 5:4434. 10.1038/ncomms5434 25026888

[24] Hinten M Maclary E Gayen S Harris C Kalantry S. Visualizing long noncoding RNAs on chromatin. Methods Mol Biol. (2016) 1402:147–64. 10.1007/978-1-4939-3378-5_12 26721489 PMC5094191

[25] Huang W Thomas B Flynn RA Gavzy SJ Wu L Kim SV. DDX5 and its associated lncRNA Rmrp modulate TH17 cell effector functions. Nature. (2015) 528:517–22. 10.1038/nature16193 26675721 PMC4762670

[26] Wu Y Hu L Liang Y Li J Wang K Chen X. Up-regulation of lncRNA CASC9 promotes esophageal squamous cell carcinoma growth by negatively regulating PDCD4 expression through EZH2. Mol Cancer. (2017) 16:150. 10.1186/s12943-017-0715-7 28854977 PMC5577767

[27] Kim J Piao HL Kim BJ Yao F Han Z Wang Y. Long noncoding RNA MALAT1 suppresses breast cancer metastasis. Nat Genet. (2018) 50:1705–15. 10.1038/s41588-018-0252-3 30349115 PMC6265076

[28] The lnCRNA NORAD Contributes to the Maintenance of Genomic Stability Cancer Discov. (2018) 8:1209 10.1158/2159-8290.CD-RW2018-154

[29] Boulland ML Marquet J Molinier-Frenkel V Moller P Guiter C Lasoudris F. Human IL4I1 is a secreted L-phenylalanine oxidase expressed by mature dendritic cells that inhibits T-lymphocyte proliferation. Blood. (2007) 110:220–7. 10.1182/blood-2006-07-036210 17356132

[30] Lasoudris F Cousin C Prevost-Blondel A Martin-Garcia N Abd-Alsamad I Ortonne N. IL4I1: an inhibitor of the CD8(+) antitumor T-cell response *in vivo*. Eur J Immunol. (2011) 41:1629–38. 10.1002/eji.201041119 21469114 PMC3472400

[31] Yue Y Huang W Liang J Guo J Ji J Yao Y. IL4I1 is a novel regulator of M2 macrophage polarization that can inhibit T cell activation via L-tryptophan and arginine depletion and IL-10 production. PLoS ONE. (2015) 10:e0142979. 10.1371/journal.pone.0142979 26599209 PMC4658051

[32] Martin C Zhang Y. The diverse functions of histone lysine methylation. Nat Rev Mol Cell Biol. (2005) 6:838–49. 10.1038/nrm1761 16261189

[33] Piunti A Shilatifard A. Epigenetic balance of gene expression by Polycomb and COMPASS families. Science. (2016) 352:aad9780. 10.1126/science.aad9780 27257261

[34] Gabrilovich DI Ostrand-Rosenberg S Bronte V. Coordinated regulation of myeloid cells by tumours. Nat Rev Immunol. (2012) 12:253–68. 10.1038/nri3175 22437938 PMC3587148

[35] Yamauchi Y Safi S Blattner C Rathinasamy A Umansky L Juenger S. Circulating and tumor myeloid-derived suppressor cells in resectable non-small cell lung cancer. Am J Respir Crit Care Med. (2018) 198:777–87. 10.1164/rccm.201708-1707OC 29617574

[36] Safarzadeh E Hashemzadeh S Duijf PHG Mansoori B Khaze V Mohammadi A. Circulating myeloid-derived suppressor cells: an independent prognostic factor in patients with breast cancer. J Cell Physiol. (2019) 234:3515–25. 10.1002/jcp.26896 30362521

[37] Safarzadeh E Orangi M Mohammadi H Babaie F Baradaran B. Myeloid-derived suppressor cells: important contributors to tumor progression and metastasis. J Cell Physiol. (2018) 233:3024–36. 10.1002/jcp.26075 28661031

[38] Haverkamp JM Smith AM Weinlich R Dillon CP Qualls JE Neale G. Myeloid-derived suppressor activity is mediated by monocytic lineages maintained by continuous inhibition of extrinsic and intrinsic death pathways. Immunity. (2014) 41:947–59. 10.1016/j.immuni.2014.10.020 25500368 PMC4272664

[39] Romagnani S. IL4I1: Key immunoregulator at a crossroads of divergent T-cell functions. Eur J Immunol. (2016) 46:2302–05. 10.1002/eji.201646617 27726138

[40] Marigo I Bosio E Solito S Mesa C Fernandez A Dolcetti L. Tumor-induced tolerance and immune suppression depend on the C/EBPbeta transcription factor. Immunity. (2010) 32:790–802. 10.1016/j.immuni.2010.05.010 20605485

[41] Ang YS Tsai SY Lee DF Monk J Su J Ratnakumar K. Wdr5 mediates self-renewal and reprogramming via the embryonic stem cell core transcriptional network. Cell. (2011) 145:183–97. 10.1016/j.cell.2011.03.003 21477851 PMC3097468

[42] Kuo CH Hsieh CC Kuo HF Huang MY Yang SN Chen LC. Phthalates suppress type I interferon in human plasmacytoid dendritic cells via epigenetic regulation. Allergy. (2013) 68:870–9. 10.1111/all.12162 23738920

[43] Kuo CH Lin CH Yang SN Huang MY Chen HL Kuo PL. Effect of prostaglandin I2 analogs on cytokine expression in human myeloid dendritic cells via epigenetic regulation. Mol Med. (2012) 18:433–44. 10.2119/molmed.2011.00193 22231731 PMC3356435

[44] Wang KC Yang YW Liu B Sanyal A Corces-Zimmerman R Chen Y. A long noncoding RNA maintains active chromatin to coordinate homeotic gene expression. Nature. (2011) 472:120–4. 10.1038/nature09819 21423168 PMC3670758

[45] Youn JI Kumar V Collazo M Nefedova Y Condamine T Cheng P. Epigenetic silencing of retinoblastoma gene regulates pathologic differentiation of myeloid cells in cancer. Nature Immunol. (2013) 14:211–20. 10.1038/ni.2526 23354483 PMC3578019

