# Supplementary material for: Lnc-C/EBPβ Modulates Differentiation of MDSCs Through Downregulating IL4i1 With C/EBPβ LIP and WDR5
Source: Front Immunol. 2019 Jul 17;10:1661. doi: 10.3389/fimmu.2019.01661 (PMC6650770; doi:10.3389/fimmu.2019.01661)
Supplement: Supplementary file 1 [file Data_Sheet_1.PDF]

Supplementary Material

1 Supplementary Figures and Tables

1.1 Supplementary Figures

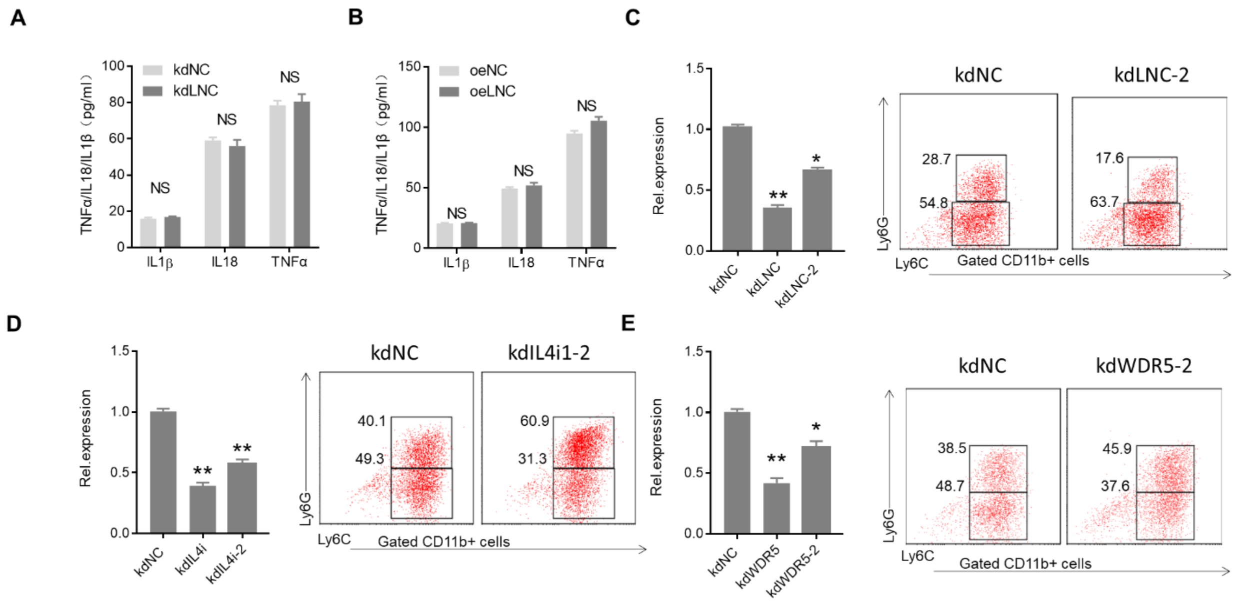

Supplementary Figure 1. Detection of shRNA or siRNA off-target effects. **(A-B)** Detection of inflammatory factors by ELISA after infection with *lnc-C/EBPβ* shRNA lentivirus (kdLNC) (A) and *lnc-C/EBPβ* over-expression lentivirus (oeLNC) (B) control lentivirus (kdNC or oeNC). **(C)** qRT-PCR of mouse *lnc-C/EBPβ* in cells transfected with different siRNA (kdLNC2) and flow cytometry of $CD11b^+ly6G^+Ly6C^-$, $CD11b^+ly6G^-Ly6C^+$ MDSCs in *lnc-C/EBPβ* knockdown MDSCs. **(D)** qRT-PCR of mouse IL4i1 in cells transfected with different siRNA（kdIL4i1-2）and flow cytometry of $CD11b^+ly6G^+Ly6C^-$, $CD11b^+ly6G^-Ly6C^+$ MDSCs in IL4i1 knockdown MDSCs. **(E)** qRT-PCR of mouse WDR5 in cells transfected with different siRNA（kdWDR5-2）and flow cytometry of $CD11b^+ly6G^+Ly6C^-$, $CD11b^+ly6G^-Ly6C^+$ MDSCs in WDR5 knockdown MDSCs. Two-tailed, paired T test was used. *P < 0.05; **p<0.05; ***p<0.001.

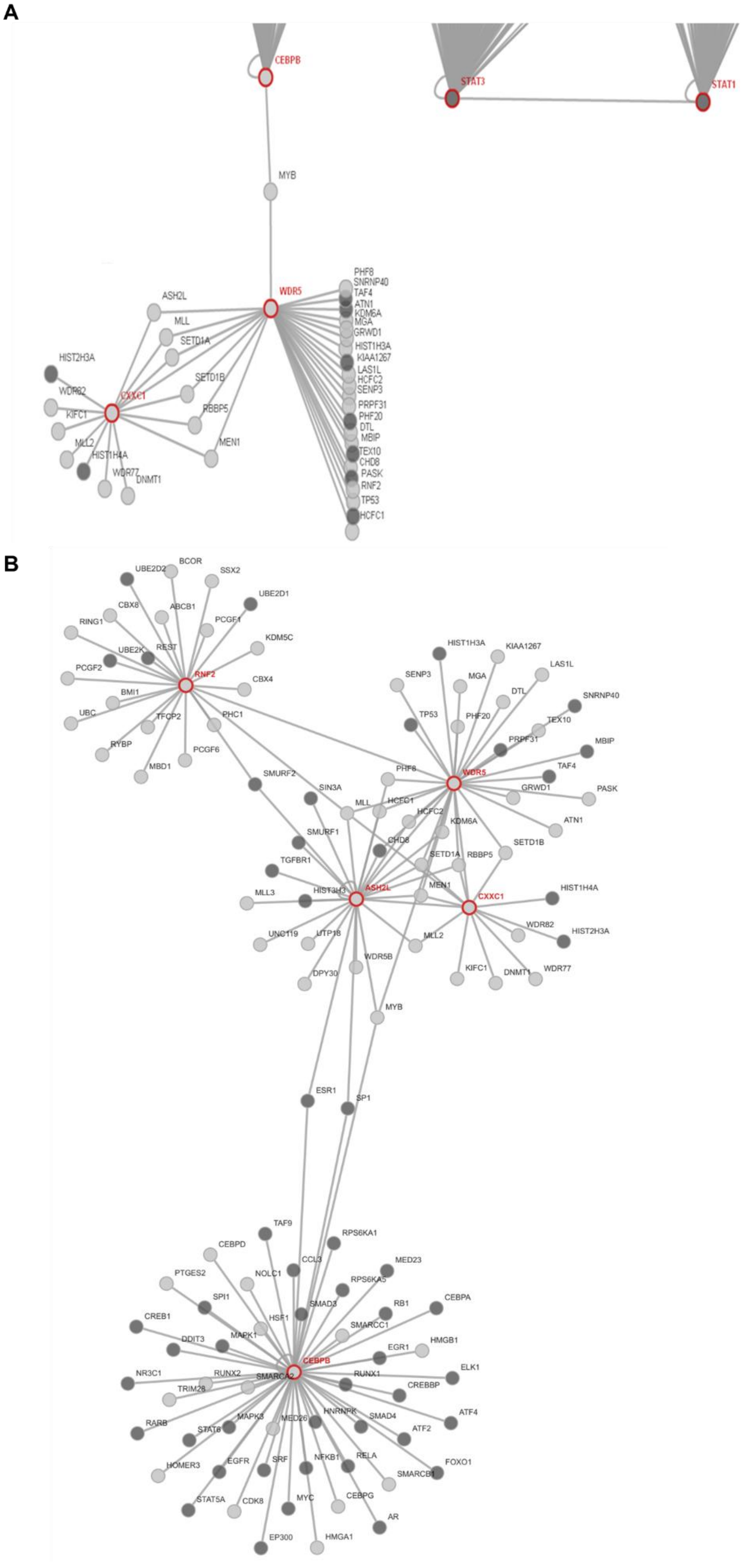
A
CEBPB
STAT3
STAT1
MYB
WDR5
ASH2L
MLL
SETD1A
SETD1B
RBBP5
MEN1
CXXC1
HIST2H3A
WDR82
KIFC1
MLL2
HIST1H4A
WDR77
DNMT1
PHF8
SNRNP40
TAF4
ATN1
KDM6A
MGA
GRWD1
HIST1H3A
KIAA1267
LAS1L
HCFC2
SENP3
PRPF31
PHF20
DTL
MBIP
TEX10
CHD8
PASK
RNF2
TP53
HCFC1
B
UBE2D2
BCOR
SSX2
UBE2D1
CBX8
ABCB1
RING1
PCGF1
KDM5C
UBE2K
REST
PCGF2
RNF2
CBX4
BMI1
UBC
TFCP2
PHC1
RYBP
PCGF6
MBD1
SMURF2
SIN3A
MLL
HCFC1
HCFC2
SMURF1
CHD8
TGFBR1
SETD1A
RBBP5
MLL3
HIST3H3
ASH2L
MEN1
CXXC1
UNC119
UTP18
MLL2
WDR5B
DPY30
MYB
ESR1
SP1
HIST1H3A
KIAA1267
LAS1L
SENP3
MGA
DTL
SNRNP40
TP53
PHF20
TEX10
PRPF31
MBIP
PHF8
WDR5
TAF4
GRWD1
PASK
KDM6A
ATN1
SETD1B
HIST1H4A
WDR82
HIST2H3A
KIFC1
DNMT1
WDR77
TAF9
RPS6KA1
CEBPD
CCL3
MED23
PTGES2
NOLC1
RPS6KA5
SPI1
SMAD3
RB1
CEBPA
CREB1
HSF1
SMARCC1
DDIT3
MAPK1
EGR1
HMGB1
CEBPB
ELK1
NR3C1
RUNX2
SMARCA2
RUNX1
TRIM28
CREBBP
RARB
MAPK3
MED26
HNRNPK
SMAD4
ATF4
STAT6
ATF2
HOMER3
EGFR
SRF
NFKB1
RELA
FOXO1
SMARCB1
STAT5A
CDK8
MYC
CEBPG
AR
EP300
HMGA1

Supplementary Figure 2. The network of CEBP/β and its interacting proteins in GM-CSF only **(A)** or IL-6 and GM-CSF **(B)** treated MDSC. GM-CSF alone and GM-CSF plus IL6 (GM+IL6) mediated MDSC were immunoprecipitated with C/EBPβ antibody, and subjected to SDS-PAGE with silver staining, and then MASS was performed. The networks of CEBP/β and its interacting proteins were done by pathway linker based on the C/EBPβ interacting proteins in GM-CSF alone and GM-CSF+IL6 (GM+IL6) mediated MDSC. Red nodes, queried proteins; Dark gray, signaling pathway member proteins; light grays, non-pathway members; Links, interactions. p-values below 0.01 indicate that signaling pathways are significantly overrepresented in the set of displayed proteins containing the queried protein (s) and their first neighbor interaction.

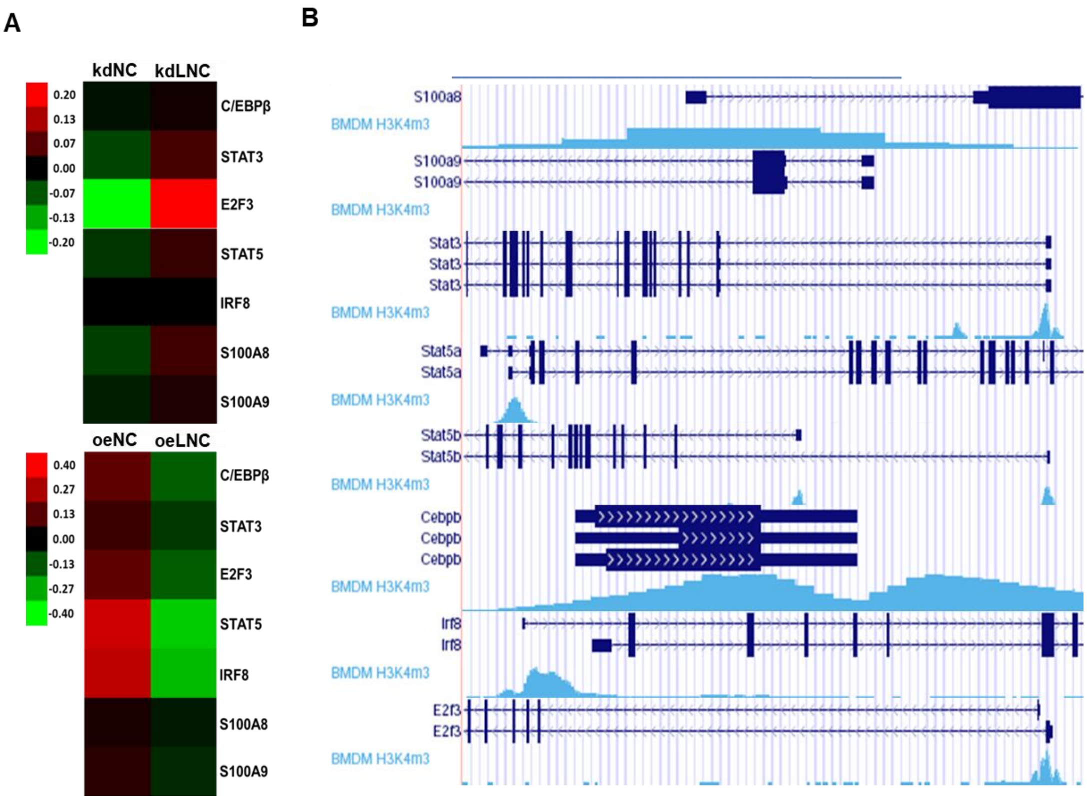

Supplementary Figure 3. MDSC associated gene expression and epigenetic modification. **(A)** Differential expression of MDSC differentiation-associated genes in *lnc-C/EBPβ* knockdown and extrogenous *lnc-C/EBPβ* treated MDSCs. Mouse bone marrow cells were transfected with *lnc-C/EBPβ* shRNA (kdLNC) or extrogenous *lnc-C/EBPβ* (oeLNC) and then cultured for 4 days in the presence of GM-CSF and IL-6. kdNC and oeNC, control lentiviruses.**(B)** Genome Browser image showing the accumulation of H3K4me3 on the promoter regions of MDSC-associated genes S100A8, S100A9, STAT3, STAT5a, STAT5b, C/EBPβ, IRF8 and E2F3.

1.2 1.2 Supplementary table S1. Primers used in this study.

**Gene symbol**	**sense primer (5'-3')**	**Antisense primer (5'-3')**
**Primers for qRT-PCR**		
GAPDH	TCAACGGCACAGTCAAGG	TACTCAGCACCGGCCTCA
lnc-C/EBPβ	GCTTGCACAGGGTATGAAGA	TCGTTTCTCTCCGTTTGAGAT
IL4i1	ATCACTCAGGGGAGGAACGAT	CGACGGAAACTCAGAAAAACC

WDR5	GAGCACAGCCCACTCCTTCC	ACTTGCCAACCATTCCCCAT
**Primers for plasmid constructs**		
LAP-V5	GCCACCATGGAAGTGGCCAACTTCTACTACGAG	GCAGTGGCCCGCCGAGGCCA
LIP-V5	GCCACCATGGCGGCCGGTTTCCCGTTCG	GCAGTGGCCCGCCGAGGCCA
LIP	GCCACCATGGCGGCCGGTTTCCCGTTCG	CTAGCAGTGGCCCGCCGAGGCCA
WDR5-V5	GCCACCATGGCCACAGAGGAGAAGAAGC	GCAGTCACTCTTCCACAGTTTGATT
WDR5	GCCACCATGGCCACAGAGGAGAAGAAGC	TTAGCAGTCACTCTTCCACAGTTTGATT
IL4i1	GCCACCATGGCTGGGCTGGCCCTGCG	TTAGGAGTGGTCCCCCACTCGGTGC
Lnc-C/EBPβ	CGGCCGCTGGAAATAGAG	CCTTAATCAGATATAATTTTATTCAC
**Primers for CHIP-PCR**		
H3K4me3 Arg1	AAGAGGAAACAGGAAGCGAAA	TGAAATACAGTCTCCAGGAAAATC
H3K4me3 COX2	TTCTCCCCATTAGCAGCCAG	GAGGGGGAAAAGTTGGTGGG
H3K4me3 NOS2	CTCAGTCTTCAACTCCCTGTAAA	GTCCATCATGAATGAGCTAACTT
H3K4me3 NOX2	GTACCCAAACCAGGTAACATAC	GCAAACAACAATAATAAAAGCC
Il4i1-H3K4me3 P0	TGTTGTCTGCCTAGCAAAGG	CAAGAGGCTGAGGTAGATGGA
Il4i1-H3K4me3 P1	GCCTTACCTTCTGTTTGTCA	GATTTATAGCCAGCAGTCTGT
Il4i1-H3K4me3 P2	AAGGCAACGGCAGAAGAAT	CAGGTCCTGATAGGAAGAGCA
Il4i1-H3K4me3 P3	AAAGACCACCATGCAGTGAG	CTGGACAGGAACAGAGCAAT
Il4i1-H3K4me3 P4	TGCAGTTAGGTCCCAGAGCC	CAGAAACAGTGCCACATCCC
Il4i1-H3K4me3 P5	TTTCCCAAAGCCAGCAAC	TAGGAGCAAGCAAGTAAGTGTC
Il4i1-H3K4me3 P6	AGGAAAGGAGTCTATCCCAATG	CCAAATCCCATGTTAGTGCC
Il4i1-H3K4me3 P7	TACTGGTGCAGAATGAACTCC	CCTTCCCTGAAGACAAGAATA
Il4i1-3001/3200	GGGGAAAAAGTGTAAAATGTC	ACATAGATCAAGTCTCACAAAGC
Il4i1-3150/3350	AGGTATGGGGAAGGAATGAGT	TTTCAAATGCTGCCTTGTAAC
Il4i1-3300/3500	TACAGAGGTCTCTAAAAGTTTTCTA	CAGCTGCTACTTAAGCAAACTTA
Il4i1-3450/3650	GGTCCTCTCAAGCCTTAAAG	CTCCTTCAGAAGCTGAACTTT
Il4i1-3600/3800	CAGTTACTGATTTGATTAAAGGG	CATCATATAAACCAGCTGTG
Il4i1-3750/4000	CTCGTGAGTGCTGGAATCATAG	AGCACTTGGGAGGCAGAGAC
**Probes used in the RNA-FISH**		
Lnc-C/EBPβ	FAM--GAGCCAGGGCAGCCTGTCAGTCAC	
NC-FAM	FAM--CGGGAGCCTAGGAAGTGCATCTTTC	
**shRNAs used in this study**		
sh*Lnc-C/EBPβ*	gatccGTTCTCCGAACGTGTCACGTAATTCAAGAGATTACGTGACACGTTCGGAGAATTTTTTc	
shLnc C/EBPβ-2	gatccGGAAGATTGACACTAAGAAttcaagagaTTCTTAGTGTCAATCTTCCttttttc	
**siRNAs used in this study**		
IL4i1	GCCGCGGTGAGAATCAATA	
IL4i1-2	CCTCCAGCTTGAACCCTAT	
WDR5	GCAAGCCCACACCAGTTAA	

WDR5-2	GCCACAAGAATGAGAAGTA
